# Regulatory Effects of Companion Plants (Maize (*Zea mays*) and *Perilla frutescens*) on American Ginseng Growth and Microbiome in Root Rot-Infested Field

**DOI:** 10.3390/plants14121871

**Published:** 2025-06-18

**Authors:** Dan Luo, Dengqun Liao, Tingting Han, Changhao Ji, Chao He, Xianen Li

**Affiliations:** 1 Institute of Medicinal Plant Development, Chinese Academy of Medical Sciences & Peking Union Medical College, Beijing 100193, China; ld131838@163.com (D.L.); dqliao@implad.ac.cn (D.L.); htt6085@163.com (T.H.); 2 School of Life Sciences, Hebei University, Baoding 071002, China; jibawymea640883@126.com

**Keywords:** American ginseng (*Panax quinquefolius*), companion planting, maize (*Zea mays*), *Perilla frutescens*, rhizospheric soil microbiome, root endophytic microbiome

## Abstract

American ginseng (AG) cultivation suffers from severe diseases, requiring heavy pesticide use. This study aimed to explore whether companion planting with maize (AG-maize) or *Perilla frutescens* (AG-perilla) could enhance AG growth and alter rhizosphere/root microbiomes in a root rot-infested field. Compared to monoculture (CK), companion planting significantly improved AG growth and survival rate at wither stage, with AG-maize showing the superior efficacy- increasing root length and fresh weight, and plant height by 39.04%, 46.10%, and 48.69%, respectively, while raising survival rate from 1.51% to 14.54%. Microbial analysis revealed that companion planting increased microbiome diversity and network complexity. At green fruit stage, AG-perilla increased rhizosphere fungal Chao1 index by 42.6%, while AG-maize and AG-perilla elevated endophytic fungal Shannon indices by 46.68% and 74.84%, respectively. At wither stage, AG-maize notably enriched beneficial microbes (e.g., soil *Pseudomonas* +108.49%, *Bacillus* +200.73%) while reducing pathogens (soil *Fusarium* −20.04%, root endophytic *Alternaria* −54.55%). Structural equation model indicated AG-maize improved AG survival via core species-driven antibiosis and nutrient regulation, with keystone species *Lysobacter* sp. RHLT3-4 and *Verrucomicrobium* sp. IMCC25902 significantly correlating with AG health. The AG-maize system fostered synergistic microbial networks, enriching beneficial taxa and suppressing pathogens. These findings provide a foundation for developing eco-friendly disease management and high-yield AG cultivation strategies.

## 1. Introduction

American ginseng *(Panax quinquefolius* L., Xi yangshen in Chinese) is a highly valuable perennial herb with a wide range of pharmacological effects such as immunomodulation, antitumor, antifatigue, antioxidation and radioprotection [1]. Although wild American ginseng roots are believed to be more potent and possess a much higher price, due to its scarcity and 10–20 years’ growth period, American ginseng roots in the world market are supplied mainly from cultivated American ginseng, which are generally harvested after 3–4 years grown in the field-shaded condition or after 6–9 years under the wood-grown environment [2]. After the successful introduction from North America into China in the 1970s, China has become the third major AG-producing country after Canada and America. AG plantation and trades become one of major agricultural income sources in Jilin, Liaoning, Wendeng city, Shandong province and Liuba county, Shananxi province. Wendeng farmers grew about 3700 hectares of AG in 2020 and provided 7500 tons of AG roots, accounting for 70% of the total yield in China [3].

Due to its severe continuous cropping obstacle that still lasts even 10 years after the first-cropping AG [4], one prerequisite for the successful cultivation of AG plants is to use the land where AG has not been planted previously. Nonetheless, AG diseases such as root rot, rust spot, damping off, anthracnose still occur seriously in the newly planted AG soil under adverse climates within the long cultivation years [5,6,7,8], resulting in a low survival rate of AG and a large yield loss [8,9]. Microbiome studies revealed that the occurrence of AG diseases was highly related to the increased fungal pathogens and the decreased beneficial bacteria in the rhizosphere of American ginseng [10,11,12,13]. Some effective agronomic measures to recover the balance of soil microbial communities and reduce the incidence of AG diseases were implemented, including application of fungicides [14,15], vermicompost and biochar [16] and proper fertilizers [17], the timing and proper soil loosening and watering management to maintain a suitable soil humidity, crop rotation with maize [18] and intercropping with ryegrass and red clover [19].

American ginseng is a slow-growing shade-requiring perennial species. Except that AG growth is disturbed by the diseases occurring under adverse climates, it is also inhibited by its allelopathic substances produced by the dead aboveground stems and fibrous roots during the annual renewing growth [9]. It was reported that the diversity and composition of bacteria and fungi in the rhizosphere of AG plants varied within four years’ growth period, with the tendency to have no beneficial effects on growth continuation of AG plants [12,20]. Therefore, it is desired to intercrop AG with some plants to reverse the balance and composition of soil microbial community that is disturbed by AG cultivation. It was reported that more AG plants could survive in the soil where *Perilla frutescens* was grown previously [9] and maize rotation could reduce the replant problem of AG [18], indicating that their intercropping with AG possibly did not have the side effects on AG growth. Companion planting, a type of intercropping, aims to improve the growth of main crop and the managements of diseases, pests and weeds by leveraging plant ecological synergies. In this study, companion roles of perilla and maize on growth of ginseng plants in root rot-infested field were investigated in the first year after AG seedling transplantation. Meantime, the variations of microbial composition and diversity in the rhizospheric soil and roots of AG plants under companion planting and monoculture conditions were investigated. Furthermore, the relationships between the enhanced survival rate of AG intercropped with maize and environmental factors were analyzed to elucidate the synergistic effects of microorganisms, soil physicochemical properties, and enzyme activities on AG survival. These results will provide a valuable reference for enhancing American ginseng survival and growth performance through companion plants in disease-infested fields without pesticide application.

## 2. Results

### 2.1. Effects of Companion Plants on the Survival Rate and Growth of American Ginseng

**Figure 1:**
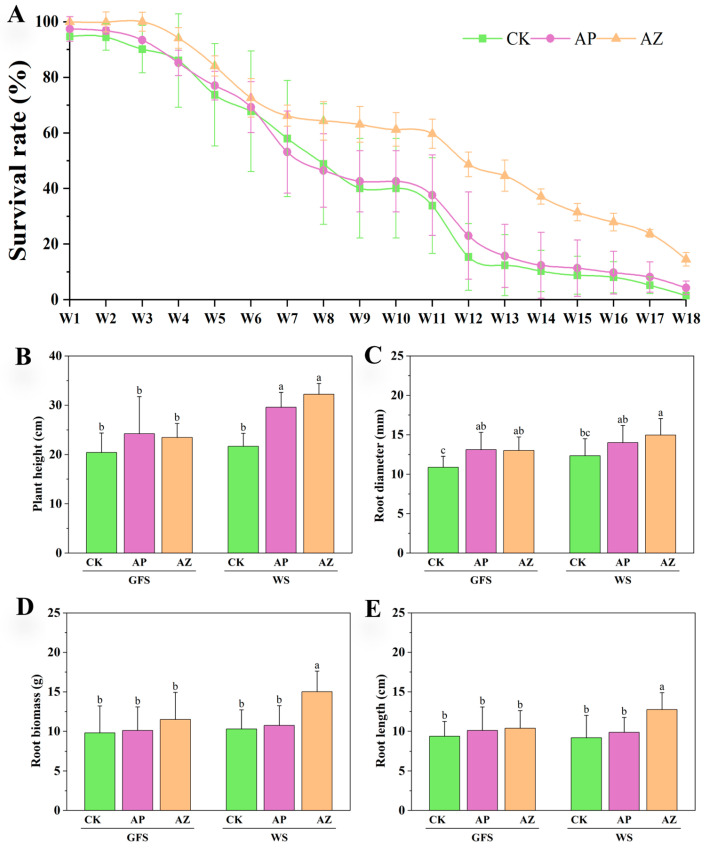
Survival rate and growth parameters of American ginseng under monoculture and intercropping conditions. CK, AP and AZ used in the graphs here and afterwards represented American ginseng (AG) plants under three cultivation systems. CK: American ginseng monoculture. AP: AG-perilla. AZ: AG-maize. In order to differentiate “AM” from the abbreviation of Arbuscular mycorrhizae, AM, we used AZ (AG-Zea mays) to replace the abbreviation of AG-maize, AM. X-axis in (**A**) referred to week after the investigation was initiated on 5 May. GFS: green fruit stage (19 July); WS: wither stage (1 September). (**B**) Plant height; (**C**) Root diameter; (**D**) Root biomass; (**E**) Root length. Different letters indicate significant differences among different treatments (*p* < 0.05).

In our experiment, 92–97% of the transplanted AG seedlings emerged in 9 plots. When companion plants perilla and maize were cultivated in early May, the survival rates of AG plants monocultured, intercropped with perilla and maize (for convenience, referred to as CK, AG-perilla, and AG-maize, separately) were 94.7%, 97.5%, and 100% (Figure 1A), indicating that some emerged AG plants began to die. Although companion planting of both perilla and maize could not prevent or greatly decrease root rot causing AG death, AG-maize showed a higher survival rate than AG-perilla and monocultured AG especially from the 11th week (mid-July) till the last sampling time (early September). The survival rate of AG-maize was about 59.7% at green fruit stage (our first sampling) while the survival rates of CK and AG-perilla were 40.0% and 42.6%, separately. The survival rates of AG-perilla were just slightly higher than CK over the growth period (*p* ≥ 0.05), indicating companion planting of perilla plants could not slow down the death of AG plants under natural condition, without fungicide use. At the experimental end, AG survival rate in maize-intercropped system (14.54%) was significantly higher than CK (1.51%,) and AG-perilla (4.25%).

Between two companion plants, only maize promoted the growth of AG plants (Figure 1B–E). At green fruit stage, AG-perilla and AG-maize treatments exhibited higher plant height, fresh root biomass, root length, and root diameter compared to CK, with ranges of 20.39–24.22 cm, 9.80–11.49 g, 9.37–10.38 cm, and 10.88–13.11 mm, respectively. At the wither stage, companion treatments significantly enhanced plant height, root length, fresh root weight, and root diameter. AG-maize demonstrated the most pronounced growth-promoting effects, increasing plant height, root diameter, fresh root biomass, and root length by 48.69%, 21.33%, 46.10%, and 39.04% respectively, relative to CK.

### 2.2. Effects of Companion Plants on Soil Physicochemical Properties and Enzymatic Activities

**Figure 2:**
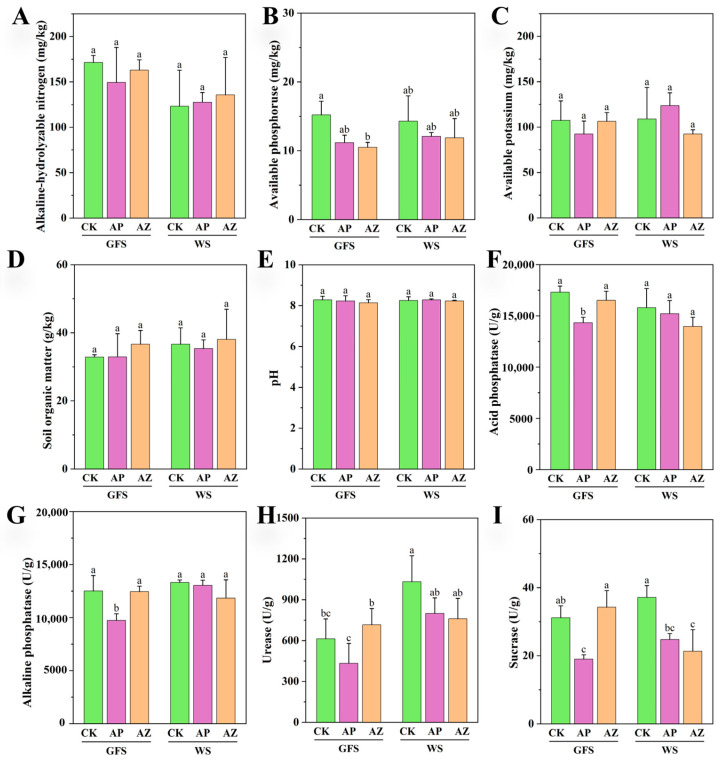
Physicochemical properties and enzymatic activities of American ginseng rhizospheric soil. Different letters on the standard error in the subgraph indicated the significant difference of the measurements among two stages under the same cultivation system or at the same stage under two cultivation systems. (**A**) Alkaline-hydrolyzable nitrogen; (**B**) Available phosphoruse; (**C**) Available potassium; (**D**) Soil organic matter; (**E**) pH; (**F**) Acid phosphatase; (**G**) Alkaline phosphatase; (**H**) Urease; (**I**) Sucrase.

The fallow soil before planting contained 149.67 mg/kg available nitrogen, 15.00 mg/kg available phosphorus, 107.67 mg/kg available potassium, and 33.93 mg/kg organic matter. Soil pH was 8.38. Our results revealed that soil pH and the content of nutrients in the AG rhizosphere did not change significantly during the growth under the same cultivation condition (Figure 2A–E), indicating that the limit nutrients sufficed for AG’s slow growth. Except for significantly lower available phosphorus in AG-maize versus CK at green fruit stage (*p* < 0.05), AG-perilla and AG-maize showed the similar physiochemical properties to the control. This suggests that intercropping perilla or maize did not complete for nutrients with AG plants.

The activities of four enzymes in the AG rhizosphere were shown in Figure 2F–I. Generally, activities remained stable across cultivation systems or growth stages under the same cultivation system. Only acid phosphatase (AG-maize), alkaline phosphatase (AG-perilla), urease (AG and AG-perilla), and sucrase (AG-maize) showed the time-varying activities within cultivation system (*p* < 0.05). At green fruit stage, intercropping perilla significantly decreased three enzymes versus CK: acid phosphatase by 17.16%, alkaline phosphatase by 22.39%, and sucrase by 38.93%. Urease activity in AG-perilla rhizosphere was also lower than CK (not significant). At wither stage, only sucrase activity were significantly lower in AG-perilla and AG-maize than CK, by 33.22% and 42.54%, separately.

### 2.3. Effects of Companion Plants on the Diversity of Microbial Community in AG Rhizosphere and Root

**Figure 3:**
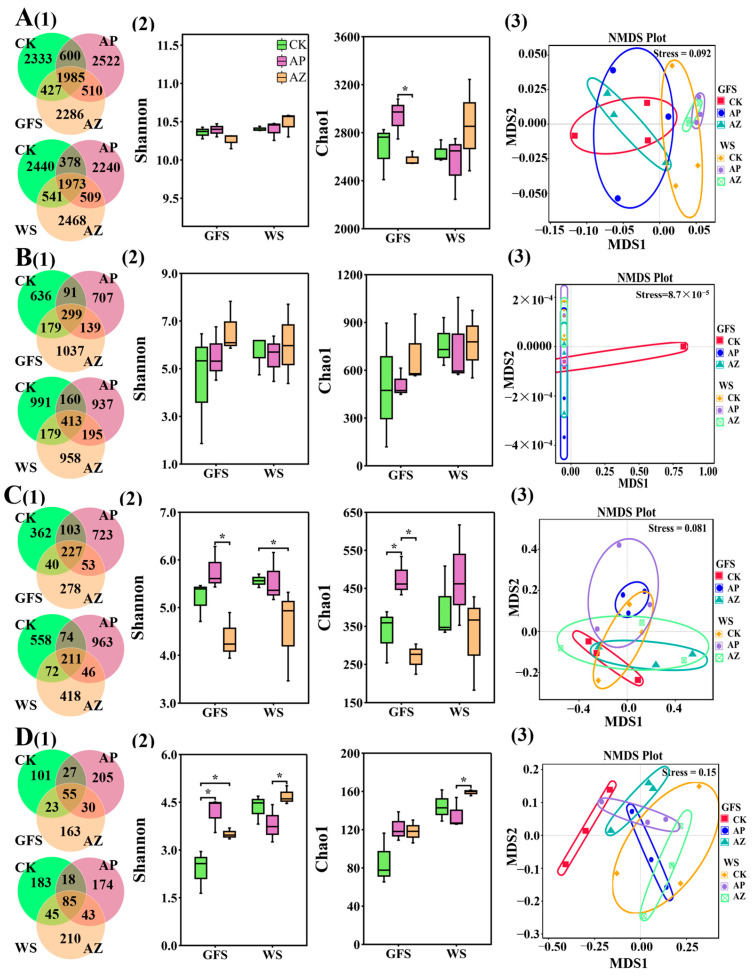
ASV distribution and microbial diversity of AG rhizosphere and root under different cropping systems. (**A**(1),**B**(1),**C**(1),**D**(1)) Venn diagrams showing ASVs shared or unique to CK, AG-perilla (AP), and AG-maize (AZ). (**A**(2),**B**(2),**C**(2),**D**(2)) Shannon and Chao1 indices showing α-diversity of microorganisms among CK, AG-perilla (AP), and AG-maize (AZ). (**A**(3), **B**(3), **C**(3),**D**(3)) NMDS plots showing β-diversity of microorganisms among CK, AG-perilla (AP), and AG-maize (AZ). (**A**) rhizosphere bacteria; (**B**) root bacteria; (**C**) rhizosphere fungi; (**D**) root fungi. * Significant difference in microbial α-diversity between two comparisons.

To assess the effects of companion planting with perilla and maize on AG rhizospheric and endophytic microbiota, amplicon sequencing was conducted to investigate the composition and diversity of bacteria and fungi in AG rhizosphere and roots at green fruit and wither stages under monoculture and intercropping conditions. The number of effective tags for individual samples was listed in Table S1. Rarefaction curves of ASVs (Figure S1) confirmed that both bacterial (16S) and fungal (ITS) sequencing data provided sufficient sequencing depth and coverage to analyze microbial diversity in AG rhizosphere and roots. AG rhizosphere amplicon sequencing generated 12,533 bacterial ASVs (Figure 3A) and 2972 fungal ASVs (Figure 3C), while AG root sequencing yielded 4514 bacterial ASVs (Figure 3B) and 871 fungal ASVs (Figure 3D). Venn diagrams showed that most ASVs were unique to specific cultivation systems, indicating that cropping systems greatly influenced microbial composition in AG rhizosphere and roots. For example, 66.97% of 10,663 rhizosphere bacterial ASVs at green fruit stage were unique to single cultivation systems (Figure 3A(1)). 1985 ASV (18.62% of rhizosphere bacteria) were common across CK, AG-perilla and AG-maize at green fruit stage. Approximately 5% microbial ASVs coexisted in two cultivation systems. Similar patterns were observed at wither stage. Few fungi ASVs were shared among three cultivation systems. Over 75% of fungal ASVs were specific to individual cultivation systems.

Shannon and Chao1 indices (Figure 3A(2),B(2)) revealed that cropping system had no significant effect on bacterial diversity in AG rhizosphere and root; NMDS analysis ((Figure 3A(3),B(3)) also revealed that samples could not be well separated among different cropping system or under the same cropping system at different growth stage. However, Fungal diversity was influenced greatly by cropping system ((Figure 3C(2),(3),D(2),(3)). AG rhizosphere had a higher alpha-diversity under monoculture and AG-perilla condition than under AG-maize condition, especially at green fruit stage (Figure 3C(2)). Fungal alpha-diversity in AG root was increased by intercropping plants especially with maize (Figure 3D(2)). Meantime, fungal alpha-diversity in AG root monocultured or intercropped with maize showed a time-increasing trend. (Figure 3D(2)). NMAS plot also revealed fungi community in AG rhizosphere or root was affected by intercropping plant and also by the growth stage (Figure 3C,D(2)).

### 2.4. Effects of Companion Plants on Microbial Composition in AG Rhizosphere and Root

As illustrated in Figure S2, the relative abundance of microbial composition in AG rhizosphere and root endophytes at phylum level was affected by cropping systems, especially at wither stage. At green fruit stage, companion plants had the minimal impact on rhizosphere bacterial phyla compared to rhizosphere fungi and root bacteria/fungi. At wither stage, *Acidobacteriota* (24.3%) in AG-perilla rhizosphere increased significantly compared to the AG, while *Actinobacteriota* and *Chloroflexi* decreased in AG-perilla and AG-maize rhizospheres (Figure S2A). At green fruit stage, *Proteobacteria* increased in roots of AG-perilla (84.61%) and AG-maize (76.03%) versus CK; *Firmicutes* decreased in AG-perilla (4.42%) and AG-maize (5.87%) versus CK (29.4%) (Figure S2B). At wither stage, *Proteobacteria* (60.16–70.05%) and *Actinobacteriota* (6.32%→11.44%) and *Chloroflexi* (9.54%→13.26%) in AG root were altered by companion planting. At green fruit stage, Rhizosphere fungi phyla *Mortierellomycota* (27.09% in AG-perilla) and *Ascomycota* (72.58% in AG-maize) increased, while *Basidiomycota* decreased in both companion systems (Figure S2C). At wither stage, AG-perilla decreased *Ascomycota* and *Basidiomycota* but increased *Mortierellomycota* compared to AG. Meanwhile, AG-maize reduced *Mortierellomycota* (16.63%) and increased *Ascomycota* (63.18%) at wither stage (Figure S2C). In terms of endophytic fungi phyla changes, at green fruit stage, AG-perilla decreased *Ascomycota* and *Mortierellomycota* but increased *Basidiomycota* (4.7%); AG-maize elevated *Ascomycota* (93.45%) and *Basidiomycota* (3.96%) while reducing *Mortierellomycota* (0.57%). At wither stage, AG-perilla increased *Ascomycota* (87.17%) and *Mortierellomycota* but reduced *Basidiomycota* (1.81%); AG-maize significantly increased *Basidiomycota* (14.68%) (Figure S2D).

**Figure 4:**
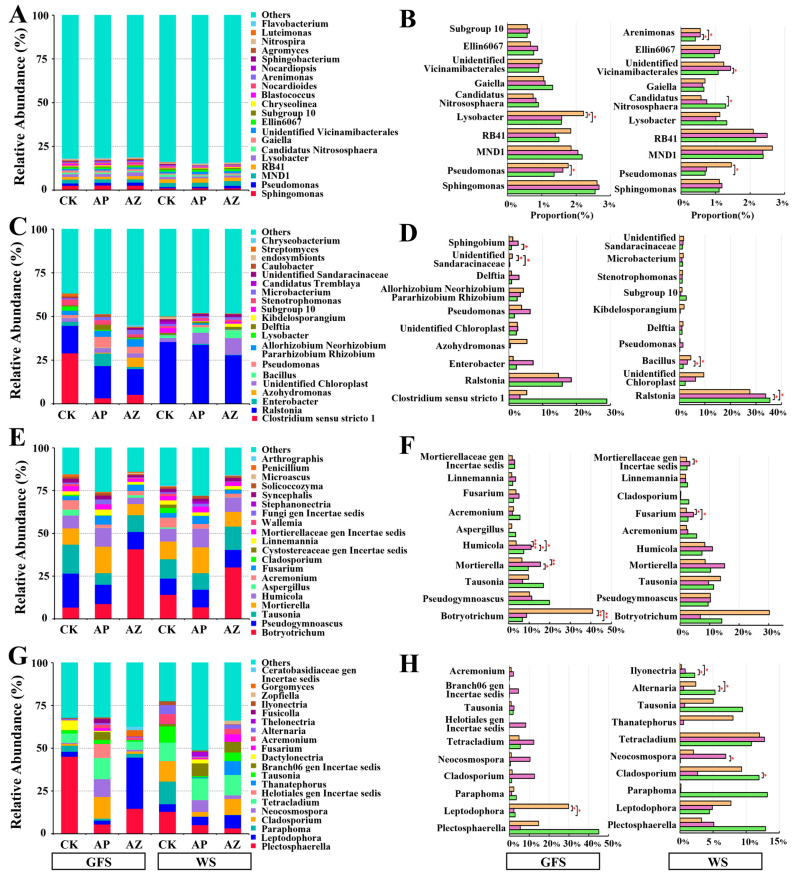
Changes of microbial genera in AG rhizosphere and root under different cropping systems. (**A**,**C**,**E**,**G**) relative abundance of the top 20 dominant microbial genera. (**B**,**D**,**F**,**H**) relative abundance s of representative genera. (**A**,**B**) rhizosphere bacteria, (**C**,**D**) root bacteria, (**E**,**F**) rhizosphere fungi, (**G**,**H**) root fungi. * *p* < 0.05, ** *p* < 0.01, *** *p* < 0.001 indicated the significant difference of microbial genus between two comparisons.

Similarly, AG rhizosphere fungi and root bacteria/fungi at genus level were greatly altered by companion planting (Figure 4A,C,E,G). Variations in microbial genera exhibited significant dependencies on AG growth stages and companion plant identity (Figure 4B,D,F,H). At green fruit stage, *Lysobacter* increased by 41.11% in AG-maize rhizosphere than CK; *Arenimonas* increased in AG-maize (33.24%) and AG-perilla (34.64%) versus CK. *Pseudomonas* increased by 30.40% (green fruit stage) and 108.49% (wither stage) in AG-maize (Figure 4B). For root bacteria, the dominant genera comprised *Clostridium sensu stricto 1*, *Ralstonia*, *Enterobacter*, *Azohydromonas*, unidentified *Chloroplast*, *Bacillus*, and *Pseudomonas* (Figure 4C). At green fruit stage, AG-perilla and AG-maize reduced *Clostridium sensu stricto 1* by 88.62% and 81.85%, respectively, relative to CK; AG-perilla increased *Enterobacter* (7.15%) and *Pseudomonas* (6.29%), whereas AG-maize elevated *Azohydromonas* and *Pseudomonas*. At wither stage, AG-maize significantly reduced *Ralstonia* by 22.12%; both treatments substantially enriched *Bacillus* (AG-maize: +200.73%; AG-perilla: +112.35%) (Figure 4D). In terms of rhizosphere fungi, at green fruit stage, compared to CK, AG-perilla significantly increased *Humicola* (10.96%) and *Mortierella* (15.54%) (*p* < 0.05); AG-maize elevated *Botryotrichum* (8.75%; *p* < 0.01) but reduced *Pseudogymnoascus*, *Mortierella*, and *Aspergillus* (Figure 4F). At wither stage, AG-perilla and AG-maize both decreased *Cladosporium* and *Verticillium* (Figure 4E). Other fungal genera showed similar changing trends in two companion systems as green fruit stage (Figure 4E). For root fungi (Figure 4G), at green fruit stage, AG-perilla and AG-maize reduced *Pseudogymnoascus* and *Dactylonectria*—compared to CK; AG-perilla increased *Cladosporium*, *Neocosmospora*, and *Tetracladium*, whereas AG-maize significantly enhanced *Leptodophora* abundance. At wither stage, both companion plants decreased *Paraphoma*, *Alternaria*, *Ilyonectria*, and *Tausonia* (Figure 4G). Notably, *Ilyonectria* was reduced by 90.01% (AG-maize) and 63.45% (AG-perilla) compared to AG. AG-perilla significantly reduced *Cladosporium*, while AG-maize increased *Leptodophora* abundance (Figure 4H).

### 2.5. Effects of Companion Plants on Microbial Co-Occurrence Networks in AG Rhizosphere and Root

**Figure 5:**
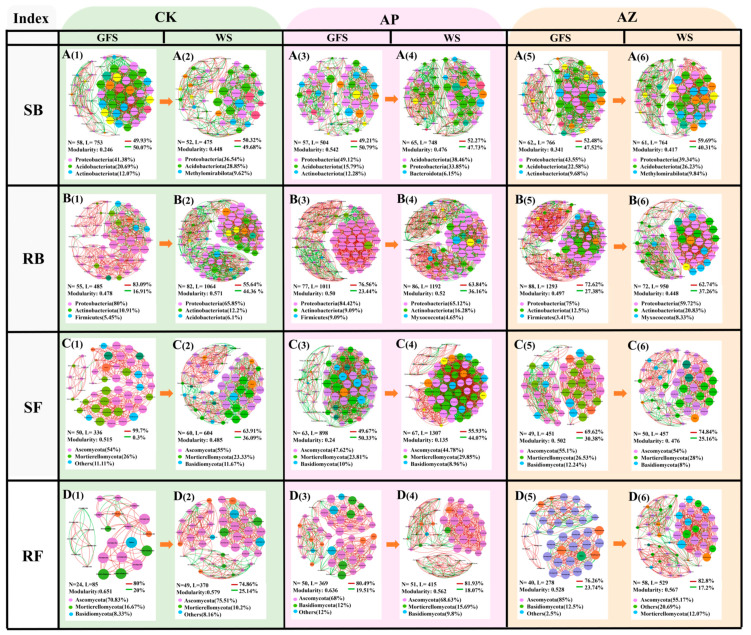
Co-occurrence networks of microbial communities in three cropping systems. (**A**) SB: rhizosphere bacteria; (**B**) RB: root bacteria; (**C**) SF: rhizosphere fungi; (**D**) RF: root fungi. (1) CK at green fruit stage; (2) CK at wither stage; (3) AG-perilla at green fruit stage; (4) AG-perilla at wither stage; (5) AG-maize at green fruit stage; (6) AG-maize at wither stage. Topological properties of microbial co-occurrence networks were listed in each subgraph. N: Number of nodes, each node represented a microbial species; L: No. of links (edges) between nodes; Edges represented significant correlations between two corresponding taxa (r > 0.6, *p* < 0.05) where Red and green indicated positive and negative correlations, respectively. Percentage of positive and negative correlations was indicated behind red and green line. The nodes of top 3 phyla were colored. Their percentage in all the phyla were showed in ().

To investigate companion planting effects on microbial interactions, bacteria/fungi co-occurrence networks were constructed using ASV levels (Figure 5). The results showed that the bacterial and fungal networks exhibited different co-occurrence patterns among individual cropping systems. In rhizosphere bacterial networks, at green fruit stage, AG-perilla and AG-maize increased the relative abundance of *Proteobacteria* compared to CK; AG-maize additionally enhanced *Acidobacteriota*. Notably, AG-perilla network had the modularity > 0.4, indicating a greater niche diversity and functional complexity (Figure 5A(1),(3),(5)). At wither stage, AG-perilla significantly increased *Acidobacteriota* and *Bacteroidetes* in rhizosphere bacterial networks, while AG-maize elevated *Proteobacteria* abundance, compared to CK. Both companion systems had more nodes, edges and positive interactions in their networks (Figure 5A(2),(4),(6)). AG-perilla exhibited the highest mutualistic interactions (positive correlations), followed by AG-maize and then CK. These results suggested that companion planting could enhance the stability of bacterial communities in AG rhizosphere.

In root bacterial networks, compared to AG monoculture, AG-perilla increased the abundances of *Proteobacteria* and *Firmicutes* at green fruit stage, while AG-maize elevated *Actinobacteriota* abundance. Compared to monoculture (CK), AG-perilla and AG-maize networks contained 40% and 60% more nodes and 108.45% and 166.60% more edges, respectively. Competitive interactions among endophytic bacteria (quantified by negative correlations) decreased in the order AG-perilla > AG-maize > CK. (Figure 5B(1),(3),(5)). At wither stagesstage, both companion systems increased *Actinobacteriota* but reduced *Acidobacteriota*. Companion planting exerted the minimal influence on endophytic bacterial co-occurrence networks in the proportion of positive/negative correlations or node distribution in. The networks of three cropping systems at wither stage had modularity > 0.4 (Figure 5B(2),(4),(6)). These results indicated that both AG-perilla and AG-maize enhanced endophytic bacterial community stability by reinforcing mutualistic cooperation and modular network structures. Concurrently, these communities exhibited intensified competition alongside elevated functional complexity. Specifically, AG-perilla facilitated collaborative endophytic functional microbiota, while AG-maize likely drove niche differentiation of endophytic bacteria through *Actinobacteriota* enrichment.

At green fruit stage, AG-perilla rhizosphere fungal network had 26% more nodes and 167%more edges than CK, but a smaller modularity (0.24) (Figure 5C(1),(3),(5)). CK networks were dominated by positive correlations (99.7%), while AG-perilla and AG-maize networks showed higher negative correlations (50.33% and 30.38%, respectively). At wither stage, both companion systems reduced *Bacteroidota* but increased *Mortierellomycota* in rhizosphere networks compared to CK (Figure 5C(2),(4),(6)). AG-perilla decreased *Ascomycota* abundance, while AG-maize increased it. Consistent with earlier patterns, AG-perilla network had more edges (1307 vs. 604) and nodes (67 vs. 60) than CK but less modularity (0.135 vs. 0.485). Conversely, AG-maize showed less nodes and edge number, and smaller modularity but higher positive correlations (74.84%).

In root endophytic fungal networks, AG-perilla network increased the number of nodes and edges at green fruit stage and only the edges at wither stage compared to CK, while AG-maize networks increased the number of nodes and edges at both growth stages (Figure 5D). While both AG-perilla and AG-maize increased *Basidiomycota* abundance at green fruit stage, they reduced *Mortierellomycota* level (Figure 5D(1),(3),(5)). Notably, AG-perilla and AG-maize exhibited differential effects on *Ascomycota* abundance. AG-perilla showed slightly reduced *Ascomycota* abundance at both stages than CK, while it increased in AG-maize roots at green fruit stage (85%) and then decreased at wither stage (55.17%). These results demonstrated that AG-perilla and AG-maize intercropping systems reshaped fungal phylum composition and increased fungal network complexity in AG root, consequently altering fungal interaction patterns. Specifically, AG-perilla intensified competitive interactions in rhizosphere fungi while weaking modular network structures, whereas AG-maize promoted the mutualistic cooperation of fungi (positive correlations) at wither stage. This suggested that AG-perilla may strengthen niche differentiation through competitive filtering, while AG-maize favors stage-specific functional collaboration among fungi. However, they both enhanced the interaction intensity among root endophytic fungal communities.

### 2.6. Relationship of Microbial Communities and Environmental Factors

**Figure 6:**
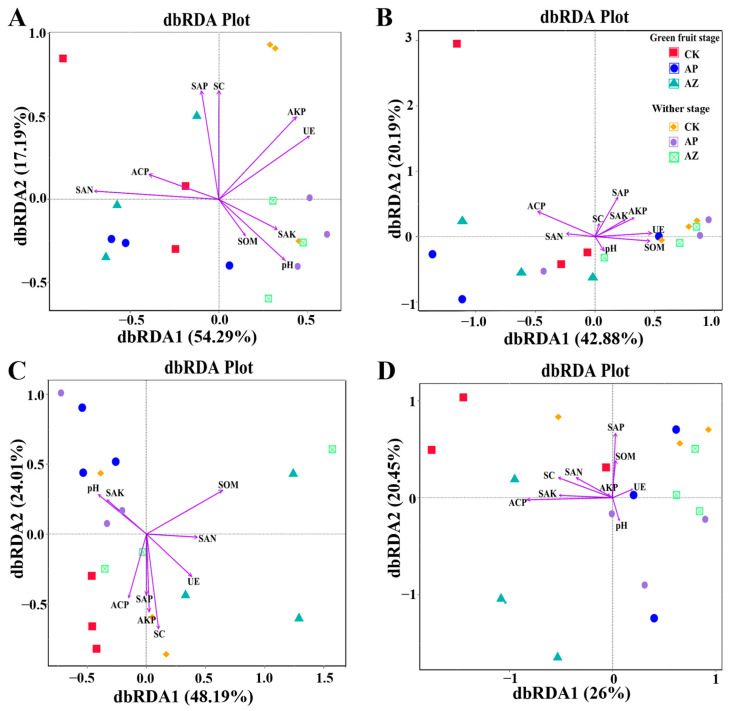
Redundancy analysis of microbial communities and environmental factors. (**A**) rhizosphere bacterial community; (**B**) root bacterial community; (**C**) rhizosphere fungi community; (**D**) root fungi community. SAN: soil Alkali-hydrolyzable nitrogen; SAP: soil available phosphorus; SAK: soil available potassium; SOM: soil organic matter; AKP: alkaline phosphatase; ACP: Acid phosphatase; UE: Urease; SC: Sucrase.

Distance-based redundancy analysis (dbRDA) revealed the correlations between soil physicochemical properties, enzyme activities, and microbial community structures (Figure 6). Rhizosphere bacterial communities were significantly influenced by Alkali-hydrolyzable nitrogen (r^2^ = 0.4075, *p* = 0.020), available phosphorus (r^2^ = 0.3518, *p* = 0.040), alkaline phosphatase (r^2^ = 0.3998, *p* = 0.021), urease (r^2^ = 0.3703, *p* = 0.038), and sucrase (r^2^ = 0.3534, *p* = 0.036), with alkali-hydrolyzable nitrogen, alkaline phosphatase, and sucrase identified as primary drivers (Figure 6A). The dbRDA1 axis explained 54.29% of the variance in these environmental-microbial relationships. No significant correlations were found between soil physicochemical properties, enzyme activities, and endophytic bacterial communities (Figure 6B). Fungal community compositions in AG rhizosphere were significantly correlated with organic matter (r^2^ = 0.371, *p* = 0.032) and sucrase activity (r^2^ = 0.397, *p* = 0.021) (Figure 6C). The dbRDA1 axis explained 48.19% of the variance. Endophytic fungal communities were significantly structured by available phosphorus (r^2^ = 0.372, *p* = 0.026) and acid phosphatase activity (r^2^ = 0.496, *p* = 0.008), with acid phosphatase activityemerging as the dominant driver. The dbRDA1 axis accounted for 26% of the observed variance in these relationships (Figure 6D).

### 2.7. Keystone Microbacterial Species Associated with Survival and Growth of AG Plant

**Figure 7:**
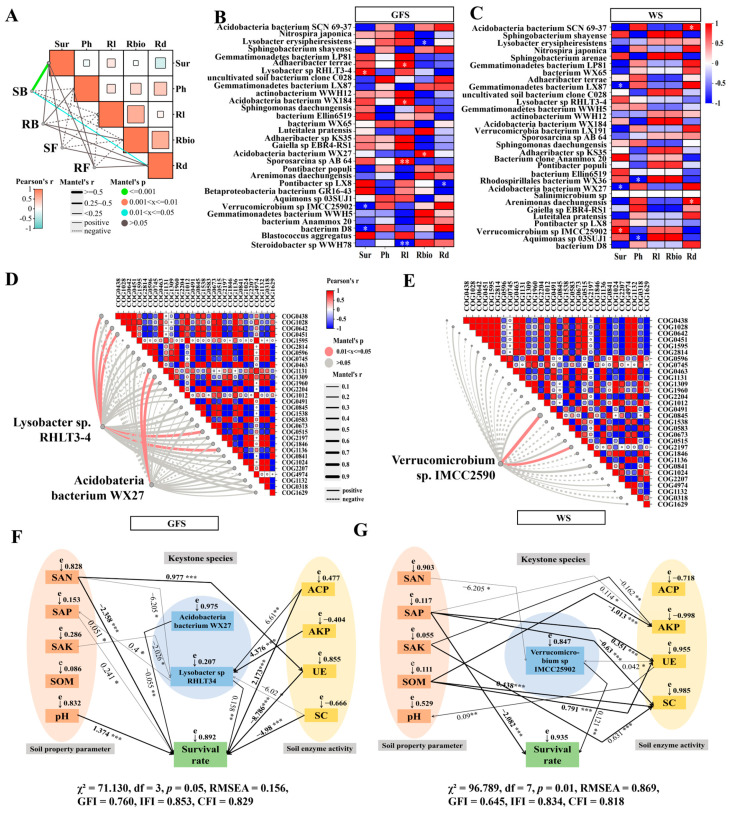
Keystone microbial species associated with survival rate and growth of American ginseng plant. (**A**) Mantel test visualizing the correlations between microbial communities with survival rate and growth of ginseng plant. Correlation heatmap of the top 30 bacterial species in AG-maize rhizosphere with survival rate and growth of ginseng plant at GFS (**B**) and WS (**C**). * on the heatmap indicated significant correlations. Mantel test visualizing the correlations between bacterial species with microbiome COG functional categories at GFS (**D**) and WS (**E**). Structural equation model (SEM) showing the effects of soil physicochemical properties, enzyme activities, and keystone bacterial species on survival rate of AG plant under maize companion planting condition at GFS (**F**) and WS (**G**) Numbers adjacent to arrows represented standardized path coefficients (* *p* < 0.05, ** *p* < 0.01, *** *p* < 0.001). Positive correlations were not marked; Negative correlations were marked “-“. Sur: survival rate. Ph: plant height. Rl: root length. Rbio: root biomass. Rd: root diameter. SAN: Alkali-hydrolyzable nitrogen. SAP: Available phosphorus. SAK: Available potassium. SOM: Soil organic matter. AKP: Alkaline phosphatase. ACP: Acid phosphatase. UE: Urease. SC: Sucrase. GFS: Green fruit stage. WS: wither stage. COG annotations were listed in Table S2.

To explore whether bacterial and fungi community in AG rhizosphere and root influenced survival rate and growth of ginseng plants, 30 top species were used in Mantel test analysis. The result revealed that ginseng survival rate was positively linked to rhizosphere bacteria composition (*p* < 0.01) (Figure 7A). Subsequent analysis of the top 30 bacterial taxa in AG-maize rhizosphere revealed 10 species exhibiting significant correlations with survival rate and growth parameters of American ginseng at the green fruit stage (Figure 7B) and 7 bacterial species at wither stage (Figure 7C). Three species showing significant positive correlations with both AG survival rate and root biomass (*p* < 0.05) were prioritized for COG functional analysis (Figure 7D,E). Mantel test analysis revealed *Lysobacter* sp. RHLT3-4 and *Acidobacteria bacterium* WX27 were positively correlated with 5 COGs and 3 COGs, respectively. *Verrucomicrobium* sp. IMCC25902 positively accossicated with AG survival rate at wither stage showed positive correlations with COG2197 and COG0845.

A structural equation model (SEM) analyis was conducted to evaluate the influences of these three bacteria and environmental factors on AG survival in maize-intercropped system (Figure 7F,G). The results showed that AG survival was highly influenced by a synergistic interplay of soil physicochemical properties, keystone microbial taxa and soil enzyme activities. At green fruit stage, alkali-hydrolyzable nitrogen, available phosphorus, acid phosphatase and *Lysobacter* sp. RHLT3-4 by were the primary drivers impacting AG’s survival rate, while available phosphorus, urease, and *Verrucomicrobium* sp. IMCC2590 influenced AG survival at wither stage.

## 3. Discussion

The ecosystem services of a companion planting system are greatly dependent on intercrops, intercropping space and pattern [21], density and sowing timing of companion plants [22,23,24,25], and interspecific interaction patterns (i.e., competition, facilitation, complementarity, and compensation). Considering the distinctive root architecture of American ginseng (featuring a taproot system with limited lateral spread and slow growth) [26,27,28] and the shallow fibrous root distribution of maize and perilla (confined to around 20 cm lateral spread), an intercropping spacing of 10 cm was maintained between each ginseng plant and its neighboring maize/perilla plants. At sampling, we observed that even when some maize and perilla fibrous roots penetrated the ginseng root zone, the 10 cm spacing preserved the structural integrity of the ginseng taproot, demonstrating negligible soil spatial competition in this intercropping configuration. Among three cropping systems, intercropping with maize at wither stage exhibited the most favorable effect on growth performance of American ginseng including the increased plant height, root length and biomass. Overall, the rhizosphere soil nutrients of American ginseng (alkaline N, available K, and soil organic matter) showed no significant differences across intercropping systems or overtime with two stages in the cropping systems. These findings align with Peng’s study demonstrating stable seasonal nutrient dynamics in ginseng rhizosphere soils [29]. This phenomenon may be attributed to: (1) minimal root intrusion (<2 mm sampling radius) from companion plants reducing nutrient competition with AG roots, and (2) the inherently slow growth rate (<7 g biomass yearly) and low nutrient demand of ginseng.

Despite the experimental field being fallow for 2 years with no prior American ginseng cultivation, root rot disease progressively developed during the growth cycle. Due to the absence of pesticide application for disease control, the survival rate of healthy ginseng plants dropped below 15% by the wither stage. However, starting in July (green fruit stage), maize intercropping (AG-maize) exhibited significantly higher healthy plant survival rates compared to both the control (CK) and perilla intercropping (AG-perilla). Conventional understanding links soil-borne disease occurrence to reduced fungal diversity [13]. However, our microbiome analysis revealed the lower fungal diversity in AG-maize during the green fruit stage compared to CK and AG-perilla, with a marked increase at the wither stage, which was partially associated with our focus on healthy ginseng plants. Meantime, pathogen abundance, rather than overall diversity, was the primary determinant of root rot severity [29]. Our results showed that both intercropping systems induced significant compositional shifts in both fungal and bacterial communities compared to AG monoculture. Among them, AG-maize markedly increased the relative abundances of PGPRs in rhizosphere soil, including *Lysobacter*, *Pseudomonas*, and *Sphingomonas* and reduced the abundance of pathogenic fungi *Fusarium* that led to root rot [5,8,11]. In contrast, AG monoculture showed elevated abundances of pathogenic fungi such as *Verticillium* and *Cladosporium*. Similar results were reported in lily-maize intercropping, where harmful fungi (*Fusarium*, *Funneliformis*) declined and beneficial genera (*Sphingomonas*, *Pseudomonas*) increased [30]. Similarly, we found that AG-perilla and AG-maize intercropping elevated the abundance of beneficial bacteria in AG roots such as *Actinobacteriota* genera, *Bacillus* and *Hyphomicrobium*, with the reduced *Ralstonia* abundance (*p* < 0.05). Endophytic microorganisms were supposed to play critical roles in root growth promotion, disease resistance, and metabolite accumulation [31,32]. It was reported that *Acidobacteriota* and *Chloroflexi* related to *P. quinquefolius* secondary metabolism were enriched in rhizosphere soil of *P. quinquefolius* intercropped with red clover and ryegrass [19]. *Hyphomicrobium* is a keystone denitrifier in methanol-driven systems [33], and *Bacillus* strains exhibit direct antibiosis, induce plant immune responses, and suppress pathogens via endospore production [34]. *Pseudomonas* spp. produce many antimicrobial secondary metabolites (phenazines, phloroglucinols, pyoluteorins, pyrrolnitrins, cyclic lipopeptides, hydrogen cyanide, and volatile organic compounds and hydrolytic enzymes (chitinases, glucanases and proteases). *Pseudomonas* genus was enriched in MCB product-treated potato rhizosphere and highly contributed to the significantly increased abundance of key enzymes in nitrogen metabolism and carbon fixation pathways [35]. *Ralstonia solanacearum* species complex (RSSC) within *Ralstonia* was a core pathogen causing bacterial wilt [36]. Compared to sole AG, AG-maize endophytic fungi contained less pathogenic fungi such as *Alternaria* and *Ilyonectria* [37] and more *Botryotrichum*. *Botryotrichum* was supposed to inhibit the growth and reproduction of pathogenic fungi by secreting secondary metabolites and occupying their ecological niches [38]. These findings suggest that companion plants could improve pathogen resistance of intercropped AG plants by promoting beneficial microbes (with antimicrobial and immune-enhancing traits), inhibiting pathogens, and optimizing plant nutrient absorption.

*Verrucomicrobium* and *Lysobacter* accounted for 55.4% and 43.8% in screened *Tremella fuciformis* polysaccharide-degrading bacterium from *Tremella fuciformis*-growing soils [39]. They were supposed to secrete extracellular carbohydrate-metabolic enzymes such as glucanase, xylanase, and mannanase involving polysaccharides degradation. Our study identified *Lysobacter* sp. RHLT3-4 and *Verrucomicrobium* sp. IMCC25902 were significantly positively correlated with the survival and growth of American ginseng intercropped with maize, where *Lysobacter* sp. RHLT3-4 acted at green fruit stage and *Verrucomicrobium* sp. IMCC25902 functioned at wither stage as a dominant beneficial bacterium. At green fruit stage, *Lysobacter* sp. RHLT3-4 operated through dual pathways: a direct path (*Lysobacter*→survival rate, *p* < 0.01), likely linked to its antimicrobial activity, and an indirect path aligned with enriched sugar metabolism and fatty acid biosynthesis pathways, indicating that *Lysobacter* sp. RHLT3-4 possibly suppressed phytopathogens and participated in nutritent metabolism to promote AG growth. Studies confirmed that many *Lysobacter* spp. suppress pathogen proliferation via secretion of chitinase [40,41] and antifungal compounds [42,43]. *Lysobacter* genus was also enriched in MCB-treated potato rhizosphere and highly contributed to the significantly increased abundance of key enzymes in nitrogen metabolism and carbon fixation pathways [35]. *Verrucomicrobium* species are cosmopolitan in rhizosphere soil but also found in roots [44]. Four novel *Verrucomicrobium* species isolated from rice roots was shown to promote root growth. Phosphorus fertilization improved rhizosphere conditions, growth, and secondary metabolite accumulation in American ginsen [45]. Na et al. found that soil AP and AK significantly positively correlated with ginseng health [46]. It showed that *Verrucomicrobia* had the indirect effect on soil phosphorus metabolism [47]. Our SEM result showed *Verrucomicrobium* species and available phosphorus jointly enhanced AG survival when they were intercropped with maize.

## 4. Materials and Methods

### 4.1. Plant Growth

Companion planting experiments of American ginseng with *Perilla frutescens* and maize were conducted in 2023 in a polytunnel covered with a four-needle knitted shade net at the Institute of Medicinal Plant Development, Beijing, China. Two-year-old seedlings of American ginseng were purchased from a grower in Liushou County, Hanzhong City, Shananxi province and grown in late February on double-row ridges with 100 cm width and 50 cm spacing. The row and plant spacing of American ginseng plants was 30 cm × 20 cm. The soil mulch was about 3 cm deep. Companion plants, 6-leaf *Perilla frutescens* seedlings and maize seeds, were planted in early May on the ridges along the outside of two ginseng rows. The row and plant spacing of both companion plants was 40 cm × 20 cm. They were grown in parallel with AG plants. Nine plots, three each for American ginseng monocropping (CK), American ginseng-*Perilla frutescens* companion pair (abbreviated as AG-Perilla) and American ginseng-maize companion pair (abbreviated as AG-maize) were arranged randomly in our polytunnel. Each cropping system had three plots, each with the area 6 m^2^ (1 m × 6 m). To reduce marginal effects and interferences from the different treatments, guarding plots were adopted between any two neighboring experimental plots. All the experimental plots were managed uniformly in the field, including irrigation by drip system and manual weeding. No pesticides or fertilizers were applied throughout the entire experimental period.

### 4.2. Survival Rate of American Ginseng Plants

The survived ginseng plants with normal aboveground parts were counted weekly during the growth period, from May 5 till early September when AG began to wither. The survival rate at each time-pointing was calculated as follows: number of living plants/maximum number of emerged seedlings.

### 4.3. Plant and Soil Sampling

American ginseng plant and rhizosphere soil samples were collected at green fruit stage (19 July) and wither stage (1 September). Six American ginseng plants in each replicate ridge were harvested to collect the rhizosphere soil and measure their root length, root fresh weight, root diameter and plant height. S-shaped sampling method was used to determine these six plants to be collected in a plot. The rhizosphere soil at a depth of 5–15 cm was obtained from the tightly adhered soil of root surfaces (≤2 mm) and passed through a 1 mm mesh to remove various debris and plant materials. Then, the rhizosphere soil was divided into three parts: one stored at −80 °C for microbial sequencing analysis, one stored at −20 °C for soil enzymatic activity assay and the third one dried at 37 °C for soil physicochemical properties. After root traits were investigated, roots of three ginseng plants were stored at −80 °C for endophytic root microbial sequencing analysis.

### 4.4. Soil Physicochemical Property Determination

Soil physicochemical properties were determined using the oven-dried soil. For soil pH assay, 10 g of soil were added into 25 mL CO_2_-free distilled water, stirred at 800 rpm for 1 min, and stood for 30 min. Then, soil pH was read by inserting a pH electrode head into the interface of the supernatant and the lower suspension.

Soil nutrients were determined using the methods described by Yang et al. [48]. Soil organic matter (SOM) was determined using Tyurin −180 °C oil bath titrimetric method. Briefly, 0.2 g of 0.1-mm sieved dry soil was weighed and added into the bottom of a dry hard glass test tube, followed by the addition of 5 mL of 0.8 mol/L 1/6K_2_Cr_2_O_7_ standard solution and 5 mL of concentrated H_2_SO_4_. Then, the test tube was boiled at 170~180 °C for precisely 5 min in the preheated paraffin oil bath. After cooling down, soil solution in the test tube was transferred and rinsed by distilled water into 250 mL of triangle flask. To obtain 1–1.5 mol/L H_2_SO_4_ in soil solution in the flask, the final volume was adjusted to 60–70 mL. Three drops of *o*-phenanthroline indicator was added into soil mixture. At last, 0.2 mol/L FeSO_4_ standard solution was used to titrate the left-over K_2_Cr_2_O_7_ in the soil solution after carbon oxidation. SOM (g/kg) was calculated as *C*(V_1_ − V_2_) × 0.003 × 1.724 × 1.1 × 1000/m, where, *C*, represents the concentration of FeSO_4_ titrant (here, 0.2 mol/L); V1 and V2 represent the volume of FeSO_4_ solution used to titrate blank and soil solution separately, when the solutions turned brick-red; m represents soil weight (g).

Soil alkali-hydrolysable nitrogen (SAN) was determined via the alkaline diffusion absorption method. Slightly modified from Yang et al. [48], 10 mL of 1N sodium hydroxide solution was added to the diffusion dish outer chamber; 0.005 mol/L (1/2) H_2_SO_4_ titrant was added to determine the amount of ammonia absorbed by indoor boric acid. SAN (g/kg) was calculated as *C*(V_1_−V_0_) × 14 × 1000/m, where, *C*, represents the concentration of H_2_SO_4_ titrant (here, 0.005 mol/L); V_1_ and V_0_ represent the volume of H_2_SO_4_ solution used to titrate blank and soil solution separately, when the solutions turned from blue to reddish; m represents soil weight (g).

Soil available phosphorus (SAP) was determined using Olsen method. 2.5 g of soil was extracted in 50 mL of 0.5 mol/L NaHCO_3_ solution (pH 8.5) at 200 rpm at 25 °C for 30 min. 1 g of phosphorus-free activated carbon was added in the extraction buffer to reduce humus disturbance in the subsequent colorimetrical analysis. 10 mL of the filtrate was well-mixed with 5 mL of Mo-Sb-Vc reagent. After incubation at 37 °C for 30 min, the reaction solution was quantified at 880 nm (UV-725S, Shanghai Lengguang Co., Shanghai, China).

5 g of soil was extracted in 50 mL of 1 mol/L NH_4_OAc solution at 200 rpm for 30 min. Then, the filtrate was used directly to quantify soil available potassium (SAK) on inductively coupled plasma-atomic emission spectrometry (Optima 5300DV, PerkinElmer, Waltham, MA, USA).

### 4.5. Soil Enzymatic Activity Assay

Four enzymatic activities in the soil were analyzed, including alkaline phosphatase, acid phosphatase, urease and sucrase. They were extracted and quantified following the protocols of the corresponding kits: BC0285, BC0145, BC0125, BC0245 (Beijing Solarbo Technology Co., Ltd., Beijing, China).

### 4.6. DNA Extraction, PCR and Illumina Miseq Sequencing

Total microbial DNA in the rhizosphere soil was extracted using a magnetic soil microbial DNA extraction kit (DP712, Tiangen Biotech Co., Ltd., Beijing, China), while DNA in American ginseng roots was extracted using CTAB method. DNA purity and concentration were measured with a NanoDrop 2000 spectrophotometer (Thermo Fisher Scientific, Wilmington, DE, USA), and DNA integrity was examined using 1% agarose gel electrophoresis. Bacteria/fungi fragments were amplified in PCR reaction solution containing 10 ng of genomic DNA, 0.2 μM of specific primer pairs (Table 1) and 15 μL of Phusion^®^ High-Fidelity PCR Master Mix (New England Biolabs, Ipswich, MA, USA). The PCR conditions included an initial denaturation at 98 °C for 1 min, 30 cycles of denaturation at 98 °C for 10 s, annealing at 50 °C for 30 s, and extension at 72 °C for 30 s, and a final extension at 72 °C for 5 min. PCR products were purified using magnetic beads and pooled in equal amounts. Target DNA fragments were recovered to construct amplicon sequencing libraries. Amplicon sequencing was conducted by Beijing NovaSeq Technology Co., Ltd. (Beijing, China) using the Illumina NovaSeq PE250 sequencing platform.

### 4.7. Bioinformatics Analysis

Paired- end reads were assigned to samples based on their unique barcode and truncated by cutting off the barcode and primer sequence. After trimming barcodes and primers, paired-end reads were assembled using FLASH (Version 1.2.11; http://ccb.jhu.edu/software/FLASH/, accessed on 17 November 2023) [49] to generate raw tags (RawTags). Residual primer sequences were removed using Cutadapt to eliminate interference in downstream analyses. High-quality clean tags (CleanTags) were obtained by stringent filtering of RawTags with fastp (Version 0.23.1) [50]. Chimeric sequences were detected and removed by aligning CleanTags against reference databases (Silva database (https://www.arb-silva.de/, accessed on 20 November 2023) for 16S/18S rRNA genes; UNITE database [https://unite.ut.ee/] for ITS regions, accessed on 25 November 2023), yielding final effective tags (EffectiveTags) for subsequent analyses.

The EffectiveTags were denoisd using the DADA2 module (default) or deblur algorithm within QIIME2 (Version QIIME2-202202) [51,52], yielding high-resolution Amplicon Sequence Variants (ASVs) and corresponding feature tables for downstream analyses. The taxonomies of ITS sequences and 16S rRNA genes were annotated by QIIME2 (ver. 2022202, classify-sklearn parameter), separately, mainly against Unite Database (ver. 9.0) and against Silva Database (SILVA_138.1_SSURef_NR99_tax_silva.fasta) and the dmp file from NCBI taxonomy database.

### 4.8. Statistical Analysis

The data obtained from the experiments were processed and tabulated using Microsoft Office Excel 2021. Bar charts, box plots, network diagrams, and heatmaps were created using the online graphing software CHIPLOT (https://www.chiplot.online/#, accessed on 10 February 2024). One-way ANOVA for measurements (AG growth parameters, soil physicochemical properties, and enzyme activities) were performed using SPSS 24.0 to evaluate their significant difference among three cropping systems. Alpha and beta diversity analyses based on amplicon sequence variant (ASV) abundance data were conducted in R-4.0.3. Relative abundance plots were generated using Perl 5.26.2, while heatmaps, Venn diagrams, and rarefaction curves were visualized with R-4.0.3. Co-occurrence networks were statistically analyzed in R-4.0.3 (packages: igraph, psych, Hmisc, vegan, dplyr, reshape2) and visualized using Gephi 0.10.1. Mantel tests were performed in R-4.0.3 (packages: dplyr, linkET, ggplot2) with Spearman correlation coefficients. Distance-based redundancy analysis (dbRDA) was implemented in R-4.0.3 (vegan package) to assess correlations between environmental factors and microbial community. Bray-Curtis distances were adopted. Structural equation modeling (SEM) was analyzed using AMOS 24.0, and graphical representations were prepared in PowerPoint 2021.

## 5. Conclusions

Our study demonstrated that companion planting (maize) significantly enhanced AG survival rate and growth performance in disease-infested soil, compared with sole AG and AG-perilla. We systematically characterized niche-specific microbiome profiles (diversity, taxonomic composition, and community assembly) in rhizosphere and roots of American ginseng during phenological transitions (green fruit vs. wither stages) under varied companion planting systems. The results found that both companion plants significantly enhanced the microbiome network complexity in rhizosphere and roots of American ginseng. Rhizosphere enrichment of plant growth-promoting rhizobacteria (PGPR; *Pseudomonas*, *Lysobacter*, and *Sphingomonas*) and root endosphere enrichment of beneficial taxa (*Bacillus*) were observed in AG-maize system, whereas pathogenic fungi (*Fusarium*, *Alternaria*, and *Ilyonectria*) were suppressed. In AG-maize system, the keystone species *Lysobacter* sp. RHLT3-4 and *Verrucomicrobium* sp. IMCC25902 demonstrated significant correlations with AG growth and survival. Structural equation modeling analysis revealed that AG-maize positively influenced AG survival through core species-mediated mechanisms involving antibiosis and nutrient regulation. While these findings derive from a single-year study, multi-year and multi-location validation is warranted to ensure reproducibility. Nevertheless, this work provides novel insights into yield-enhancement strategies for American ginseng cultivation and establishes a scientific foundation for systematically investigating disease resistance mechanisms in this valuable medicinal crop.

## Figures and Tables

**Table 1:** PCR primers to amplify bacteria and fungi DNA.

Microbial DNA Type	Amplicon Region	Forward Primer	Reverse Primer
Soil bacteria	16SV4	515F: 5′-GTGCCAGCMGCCGCGGTAA-3′	806R: 5′-GGACTACHVGGGTWTCTAAT-3′
Soil fungi	ITS1-5F	ITS5-1737F: 5′-GGAAGTAAAAGTCGTAACAAGG-3′	ITS2-2043R: 5′-GCTGCGTTCTTCATCGATGC-3′
endophytic bacteria	16SV57	799F: 5′-AACMGGATTAGATACCCKG-3′	1193R: 5′-ACGTCATCCCCACCTTCC-3′
endophytic fungi	ITS1-1F	ITS1-1F-F: 5′-CTTGGTCATTTAGAGGAAGTAA-3′	ITS1-1F-R: 5′-GCTGCGTTCTTCATCGATGC-3′

## Data Availability

Raw sequencing data of rhizosphere soil and endophytic root microbiomes were deposited into in the NCBI Sequence Read Archive (SRA) database (accession Nos. PRJNA1160787, PRJNA1160790, PRJNA1160806, and PRJNA1160809).

## Supplementary Materials

The following supporting information can be downloaded at: https://www.mdpi.com/article/10.3390/plants14121871/s1, Table S1: Number of effective tags used in microbiome bioinformatic analysis; Table S2: COG functional annotation information; Figure S1: Rarefaction curves of ASVs in soil samples and AG roots. A: rhizosphere bacteria. B: root bacteria. C: rhizosphere fungi. D: root fungi; Figure S2: Phylum-level abundance of microorganisms in AG rhizosphere and root under different cropping systems. A: rhizosphere bacteria, B: root bacteria, C: rhizosphere fungi, D: root fungi.
