# Supplementary material for: Regulatory Effects of Companion Plants (Maize (Zea mays) and Perilla frutescens) on American Ginseng Growth and Microbiome in Root Rot-Infested Field
Source: Plants (Basel). 2025 Jun 18;14(12):1871. doi: 10.3390/plants14121871 (PMC12196905; doi:10.3390/plants14121871)
Supplement: Supplementary file 1 [file plants-14-01871-s001.zip › plants-3655500-supplementary.pdf]

Table S1. Number of effective tags used in microbiome bioinformatic analysis.

Intercropped type	Sampling stage	Rhizosphere bacteria	Root bacteria	Rhizosphere fungi	Root fungi
CK	GFS	67697	99409	99137	76747
		72442	43624	98663	132779
		60518	86449	98158	90339
AP	GFS	85383	38008	96914	61806
		70447	56698	101778	14267
		74727	57049	95215	88809
AZ	GFS	66458	46087	101357	92116
		67362	44866	96575	91264
		61289	59251	82990	98449
CK	WS	67099	30691	96170	103429
		64893	44116	98480	25374
		63804	51419	88320	50160
AP	WS	71192	33090	97318	76496
		49135	76830	97743	89640
		63037	40022	97350	96243
AZ	WS	86429	71951	102419	90414
		62971	27715	103102	90202
		70312	43928	106899	36564

GFS: green fruit stage. WS: wither stage. CK: American ginseng monoculture. AP: American ginseng intercropped with perilla. AZ: American ginseng intercropped with maize.

Table S2. COG functional annotation information.

COG No.	Annotation
COG0438	Glycosyltransferase involved in cell wall bisynthesis
COG1028	NAD(P)-dependent dehydrogenase, short-chain alcohol dehydrogenase family
COG0642	Signal transduction histidine kinase
COG0451	Nucleoside-diphosphate-sugar epimerase
COG1595	DNA-directed RNA polymerase specialized sigma subunit, sigma24 family
COG2814	Predicted arabinose efflux permease, MFS family
COG0596	Pimeloyl-ACP methyl ester carboxylesterase
COG0745	DNA-binding response regulator, OmpR family, contains REC and winged-helix (wHTH) domain
COG0463	Glycosyltransferase involved in cell wall biosynthesis
COG1131	ABC-type multidrug transport system, ATPase component
COG1309	DNA-binding transcriptional regulator, AcrR family
COG1960	Acyl-CoA dehydrogenase related to the alkylation response protein AidB
COG2204	DNA-binding transcriptional response regulator, NtrC family, contains REC,

	AAA-type ATPase
COG1012	Acyl-CoA reductase or other NAD-dependent aldehyde dehydrogenase
COG0491	Glyoxylase or a related metal-dependent hydrolase, beta-lactamase superfamily II
COG0845	Multidrug efflux pump subunit AcrA (membrane-fusion protein)
COG1538	Outer membrane protein TolC
COG0538	DNA-binding transcriptional regulator, LysR family
COG0673	Predicted dehydrogenase
COG0515	Serine/threonine protein kinase
COG2197	DNA-binding response regulator, NarL/FixJ family, contains REC and HTH domains
COG1846	DNA-binding transcriptional regulator, MarR family
COG1136	ABC-type lipoprotein export system, ATPase component
COG0841	Multidrug efflux pump subunit AcrB
COG1024	Enoyl-CoA hydratase/carnithine racemase
COG2207	AraC-type DNA-binding domain and AraC-containing proteins
COG4974	Site-specific recombinase XerD
COG1132	ABC-type multidrug transport system, ATPase and permease component
COG0318	Acyl-CoA synthetase (AMP-forming)/AMP-acid ligase II

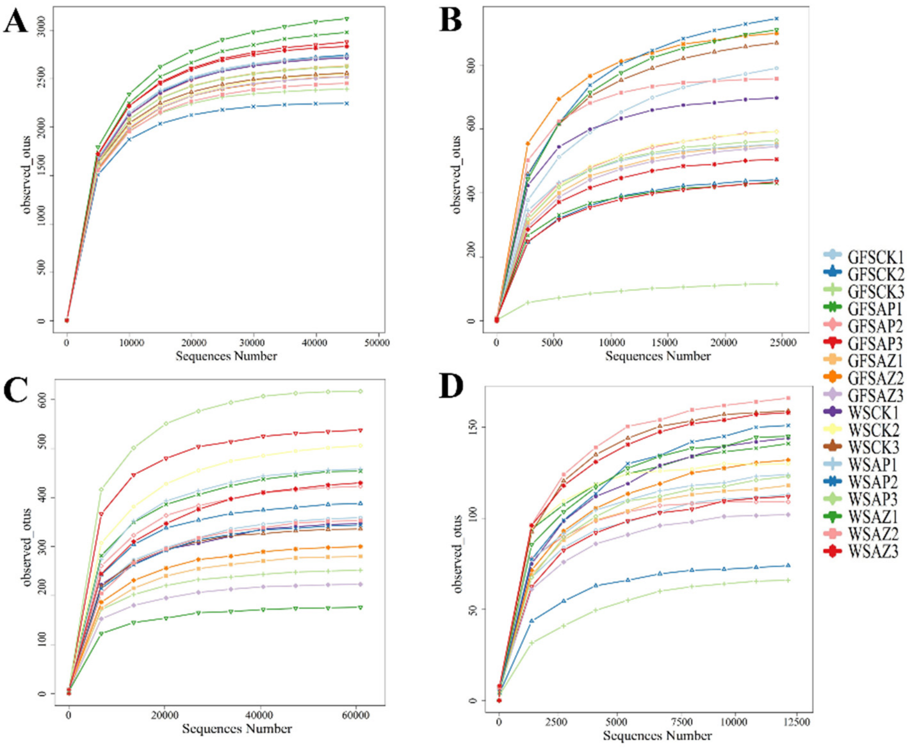

Figure S1. Rarefaction curves of ASVs in soil samples and AG roots. A: rhizosphere bacteria. B: root bacteria. C: rhizosphere fungi. D: root fungi.

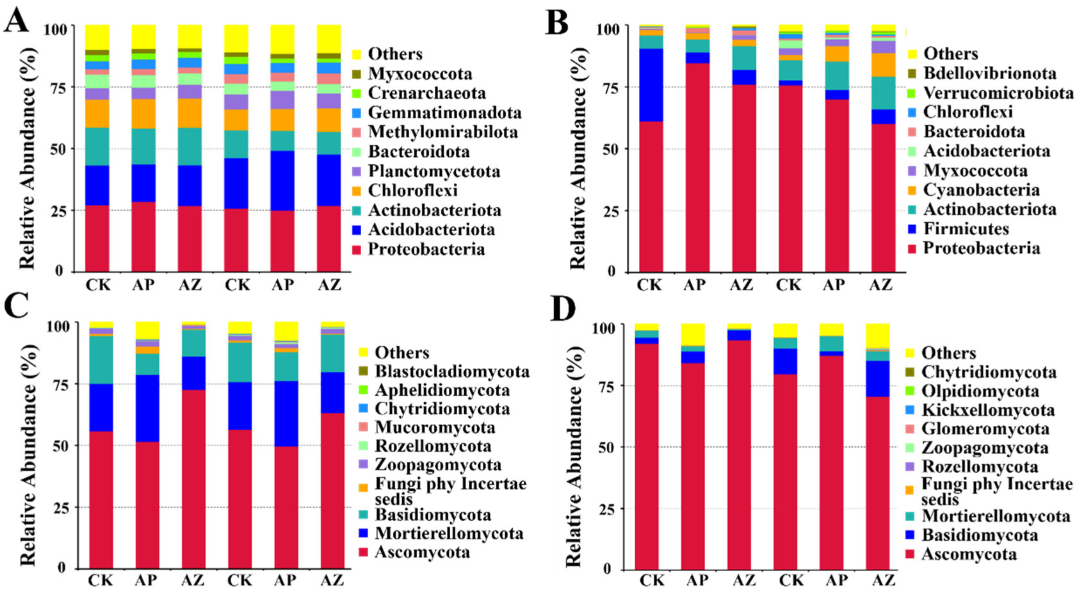

Figure S2. Phylum-level abundance of microorganisms in AG rhizosphere and root under different cropping systems. A: rhizosphere bacteria, B: root bacteria, C: rhizosphere fungi, D: root fungi.
